# TCF7L1 regulates colorectal cancer cell migration by repressing *GAS1* expression

**DOI:** 10.1038/s41598-024-63346-8

**Published:** 2024-05-30

**Authors:** Carli M. King, Wei Ding, Melanie A. Eshelman, Gregory S. Yochum

**Affiliations:** 1 Department of Surgery, Division of Colon and Rectal Surgery, The Pennsylvania State University College of Medicine, Hershey, PA 17033 USA; 2 Department of Biochemistry and Molecular Biology, The Pennsylvania State University College of Medicine, Hershey, PA 17033 USA

**Keywords:** Cancer genetics, Gene regulation

## Abstract

Dysregulated Wnt/β-catenin signaling is a common feature of colorectal cancer (CRC). The T-cell factor/lymphoid enhancer factor (TCF/LEF; hereafter, TCF) family of transcription factors are critical regulators of Wnt/β-catenin target gene expression. Of the four TCF family members, TCF7L1 predominantly functions as a transcriptional repressor. Although TCF7L1 has been ascribed an oncogenic role in CRC, only a few target genes whose expression it regulates have been characterized in this cancer. Through transcriptome analyses of TCF7L1 regulated genes, we noted enrichment for those associated with cellular migration. By silencing and overexpressing TCF7L1 in CRC cell lines, we demonstrated that TCF7L1 promoted migration, invasion, and adhesion. We localized TCF7L1 binding across the CRC genome and overlapped enriched regions with transcriptome data to identify candidate target genes. The growth arrest-specific 1 (*GAS1*) gene was among these and we demonstrated that *GAS1* is a critical mediator of TCF7L1-dependent CRC cell migratory phenotypes. Together, these findings uncover a novel role for TCF7L1 in repressing *GAS1* expression to enhance migration and invasion of CRC cells*.*

## Introduction

The Wnt/β-catenin signaling pathway controls cellular proliferation within intestinal crypts and dysregulation of this pathway drives CRC^1,2^. Mutations within components of the Wnt/β-catenin signaling pathway are found in the majority of sporadic CRCs, with up to 90% of cases containing mutations that truncate adenomatous polyposis coli (APC) or stabilize β-catenin (CTNNB1)^2–4^. These mutations lead to constitutive activation of Wnt/β-catenin signaling and initiate tumorigenesis, in part, by promoting β-catenin accumulation in the nucleus and deregulation of Wnt target gene expression^2,5^. In the nucleus, β-catenin functions as a transcriptional co-activator by interacting with TCF transcription factors^6^.

The four TCF family members, TCF7, LEF, TCF7L1, and TCF7L2, contain a conserved high mobility group (HMG) DNA-binding domain that interacts with consensus TCF binding motifs within Wnt-responsive DNA elements (WREs)^7,8^. Each family member also contains an amino-terminal β-catenin binding domain and a centrally located Groucho/transducin-like enhancer of split (Groucho/TLE) binding domain^7,8^. In addition, TCF7L1 and TCF7L2 contain PXDLS motifs within its carboxy-terminal region that interact with the corepressor C-terminal binding protein (CtBP)^7,8^. Despite conservation across DNA-binding and several protein–protein interaction domains, the TCF family members differentially regulate Wnt/β-catenin target gene expression upon binding to WREs. TCF7L2 functions as a transcriptional activator or repressor, while TCF7 and LEF1 are transcriptional activators and TCF7L1 is a transcriptional repressor^7,8^. While efforts have focused on defining the roles of TCF7L2, TCF7, and LEF1 as regulators of Wnt target genes in CRC, the role of TCF7L1 in this cancer is less-well understood.

Studies have supported an oncogenic function for TCF7L1 in a number of human malignancies, including breast cancer, leukemia, basal cell carcinoma, skin squamous cell carcinoma, and CRC^9–14^. Despite this oncogenic role, few target genes regulated by TCF7L1 have been well characterized CRC. TCF7L1 has been shown to repress expression of the Wnt antagonist, dickkopf-4 (*DKK4*), tumor suppressor ephrin type-B receptor 3 (*EPHB3*)*,* and cancer stem cell marker leucine-rich repeat containing G-protein-coupled receptor 5 (*LGR5*)^9,13,15^. Our group has also shown that TCF7L1, together with TCF7L2/β-catenin complexes, temporally regulates *MYC* expression during distinct stages of the cell cycle^16^. Identification of additional target genes whose expression are regulated by TCF7L1 is needed to further understand its oncogenic function in CRC.

Growth arrest-specific 1 (*GAS1*) encodes a glycosyl-phosphatidylinositol (GPI)-anchored plasma membrane protein that has been linked to cell cycle arrest through a p53-dependent mechanism^17–19^. Studies have supported *GAS1* functioning as a tumor or metastasis suppressor protein in a number of human cancers, including lung carcinoma, bladder carcinoma, gastric cancer, gliomas, melanoma, and CRC^20–24^. In CRC, *GAS1* negatively regulates metabolic and metastatic phenotypes and reduced levels of *GAS1* transcripts in tumors correlates with stage II and III CRC recurrence^23,25^. Despite its role as a tumor suppressor, few regulators of *GAS1* expression have been identified.

In this study, we confirm previous findings demonstrating that TCF7L1 functions as a transcriptional repressor in CRC cells^9,13^. Through RNA-sequencing (RNA-seq), we identified genes whose expression are regulated by TCF7L1 and amongst these, we noted enrichment for those associated with epithelial-mesenchymal transition (EMT). Based on this result, we demonstrated that TCF7L1 promotes CRC cell migration, invasion, and adhesion in vitro*.* We performed chromatin immunoprecipitation followed by sequencing (ChIP-sequencing) to localize TCF7L1 binding across the CRC genome. Incorporation of these binding-site data with differentially expressed genes identified 41 targets of TCF7L1-mediated repression, including *GAS1.* In rescue experiments, we demonstrated that *GAS1* is an effector of TCF7L1-mediated CRC migration and invasion. Together, these findings further support an oncogenic role for TCF7L1 in promoting CRC cell migratory properties, in part, by repressing *GAS1* expression.

## Results

### TCF7L1-regulated genes are associated with cellular migration

**Figure 1:**
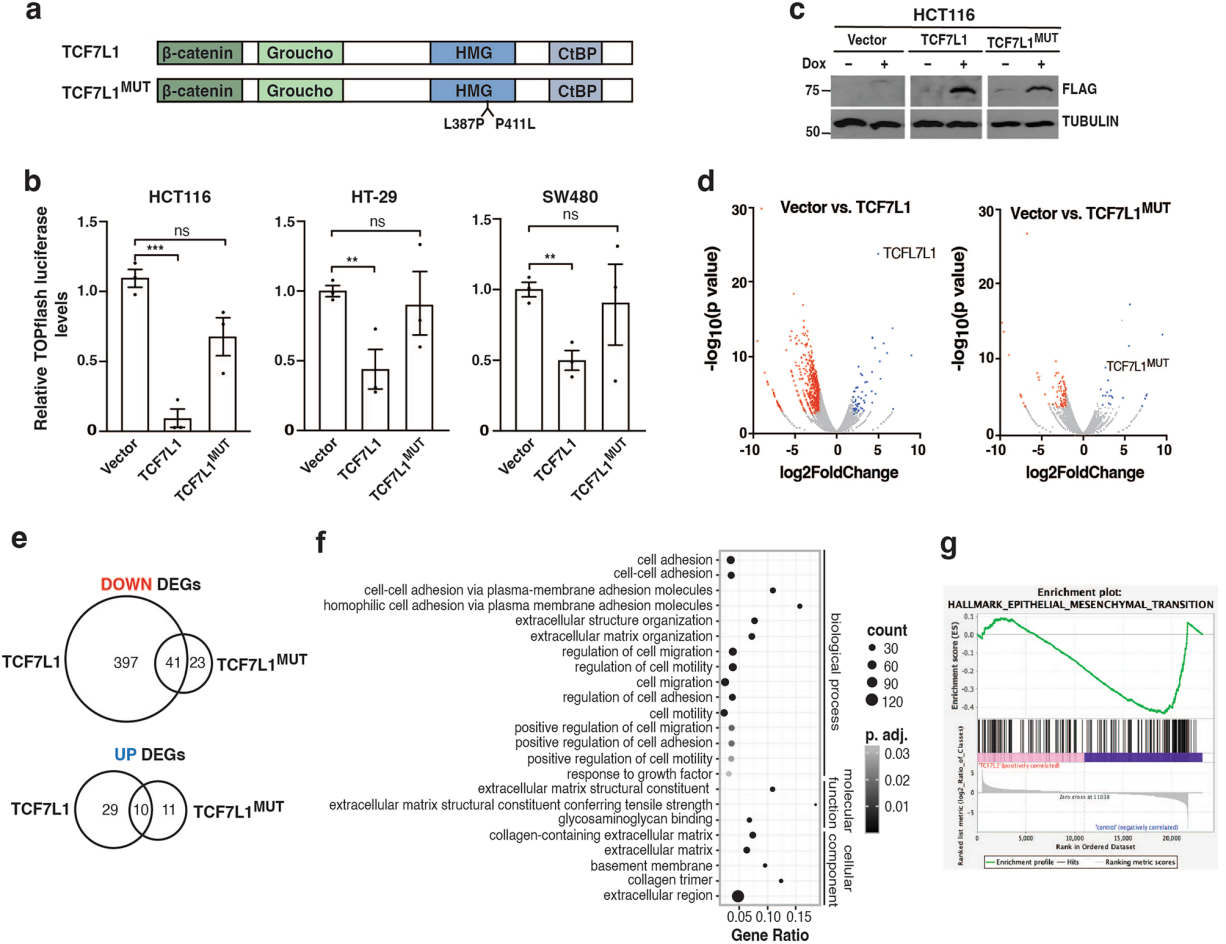
TCF7L1 regulated genes are associated with cellular migration. (**a**) Diagram of TCF7L1 with protein interaction domains and engineered point mutations in the HMG DNA binding domain indicated. (**b**) Relative luciferase levels in HCT116, HT-29 and SW480 cells transfected with a TOPflash firefly luciferase reporter plasmid and plasmids encoding wild-type TCF7L1, TCF7L1^MUT^, or vector as indicated. Luciferase values are normalized to *Renilla* luciferase levels. Data are represented as mean ± SEM (** *p* < 0.01, *** *p* < 0.001, ns, not significant). (**c**) Western blot analysis of exogenous TCF7L1 or TCF7L1^MUT^ in control (−Dox) or Dox-treated HCT116 cells. (**d**) Volcano plots of differentially expressed genes in TCF7L1-depleted and Dox-treated HCT116 cells expressing exogenous TCF7L1 or TCF7L1^MUT^. TCF7L1-depleted HCT116 cells harboring a plasmid vector served as the control. Red points are genes whose expression is significantly downregulated and blue points are genes whose expression is significantly upregulated (*p-*adj < 0.05, |log2FoldChange|> 2). (**e**) Venn diagram comparing the number of downregulated or upregulated TCF7L1 or TCF7L1^MUT^ differentially expressed genes (DEGs). (**f**) Gene ontology (GO) analysis of the 397 genes whose expression was downregulated by TCF7L1. (**g**) Gene set enrichment analysis (GSEA) of TCF7L1 regulated genes within the hallmark epithelial-mesenchymal target gene set (Nominal *p* value = 0.0; normalized enrichment score =  − 1.78).

To gain insight into the oncogenic function of TCF7L1 in CRC, we sought to identify genes whose expression it regulates. As a control for our experiments, we generated a *TCF7L1* cDNA that contains two point mutations within its HMG box DNA binding domain^11,26^. These mutations, L387P and P411L, interfere with the DNA binding capacity of TCF7L1 and we refer to TCF7L1 containing them as TCF7L1^MUT^ (Fig. 1a). In luciferase assays using the Wnt-responsive TOPflash reporter, we confirmed our previous findings that TCF7L1 functions as a transcriptional repressor in HCT116 CRC cells^9^ and found that its DNA-binding capacity was required for full activity (Fig. 1b). Similar results were also seen in two additional cell lines, HT-29 and SW480 (Fig. 1b). In our prior study, we established stable HCT116 cell lines that expressed FLAG-tagged TCF7L1 in a doxycycline (Dox)-inducible manner^15^. Stable cell lines harboring plasmid vectors or plasmids encoding FLAG-*TCF7L1*^*MUT*^ cDNAs were also established as controls. Dox treatment induced expression of FLAG-TCF7L1 or FLAG-TCF7L1^MUT^ proteins (hereafter, TCF7L1 or TCF7L1^MUT^, respectively) in stable HCT116 cells (Fig. 1c).

We next used these cells to identify TCF7L1 regulated genes. Prior to induction of exogenous TCF7L1 expression, the cells were transduced with lentivirus expressing a short hairpin RNA (shRNA) targeting *TCF7L1* transcripts previously shown to knockdown endogenous TCF7L1 proteins^9,16^. Importantly, the stable HCT116 cell lines expressed a *TCF7L1* cDNA harboring silent mutations that rendered it resistant to shRNAs ensuring that exogenous TCF7L1 would not be reduced. Following lentiviral transduction, Dox was added to induce expression of TCF7L1 or TCF7L1^MUT^ and RNAs were isolated and sequenced. We identified differentially expressed genes (DEGs; |log2fold > 2, adjusted *p *value < 0.05) from TCF7L1-expressing cells versus control cells (Dox-treated, vector) and found that 438 genes were downregulated and 39 genes were upregulated (Fig. 1d,e; Supplementary Table S1). We additionally identified DEGs from TCF7L1^MUT^-expressing cells versus control cells and found that 64 genes were downregulated and 21 genes were upregulated (Fig. 1d,e; Supplementary Table S1). As few genes were identified in TCF7L1^MUT^ cells, these results indicate that the DNA-binding capacity of TCF7L1 is required for the vast majority of target genes whose transcriptional activity it regulates. Based on these findings, we excluded down- and up- regulated DEGs from TCF7L1^MUT^ cells and focused our attention on TCF7L1 DEGs (Fig. 1e).

We next conducted gene ontology (GO) analysis on the 397 genes downregulated by TCF7L1 to gain insight on molecular pathways that are regulated by this transcription factor. This analysis identified processes associated with cell adhesion, extracellular matrix organization, cell migration, and cell population proliferation, amongst others (Fig. 1f; Supplementary Table S2). We then applied gene set enrichment analysis (GSEA) to identify genes and pathways associated with the hallmarks of cancer^27,28^ that were differentially expressed by TCF7L1 (Supplementary Table S3). We found significant enrichment (nominal *p *value < 0.05) of six hallmark sets; epithelial-mesenchymal transition (EMT) (Fig. 1g), angiogenesis, upregulated KRAS signaling, hedgehog signaling, downregulated UV response and downregulated KRAS signaling (Supplementary Fig. S1). Notably, the expression of genes comprising the Wnt/β-catenin signaling hallmark set^27^ were not significantly enriched (Supplementary Fig. S1).

### TCF7L1 promotes colorectal cancer cell migration, invasion and adhesion

**Figure 2:**
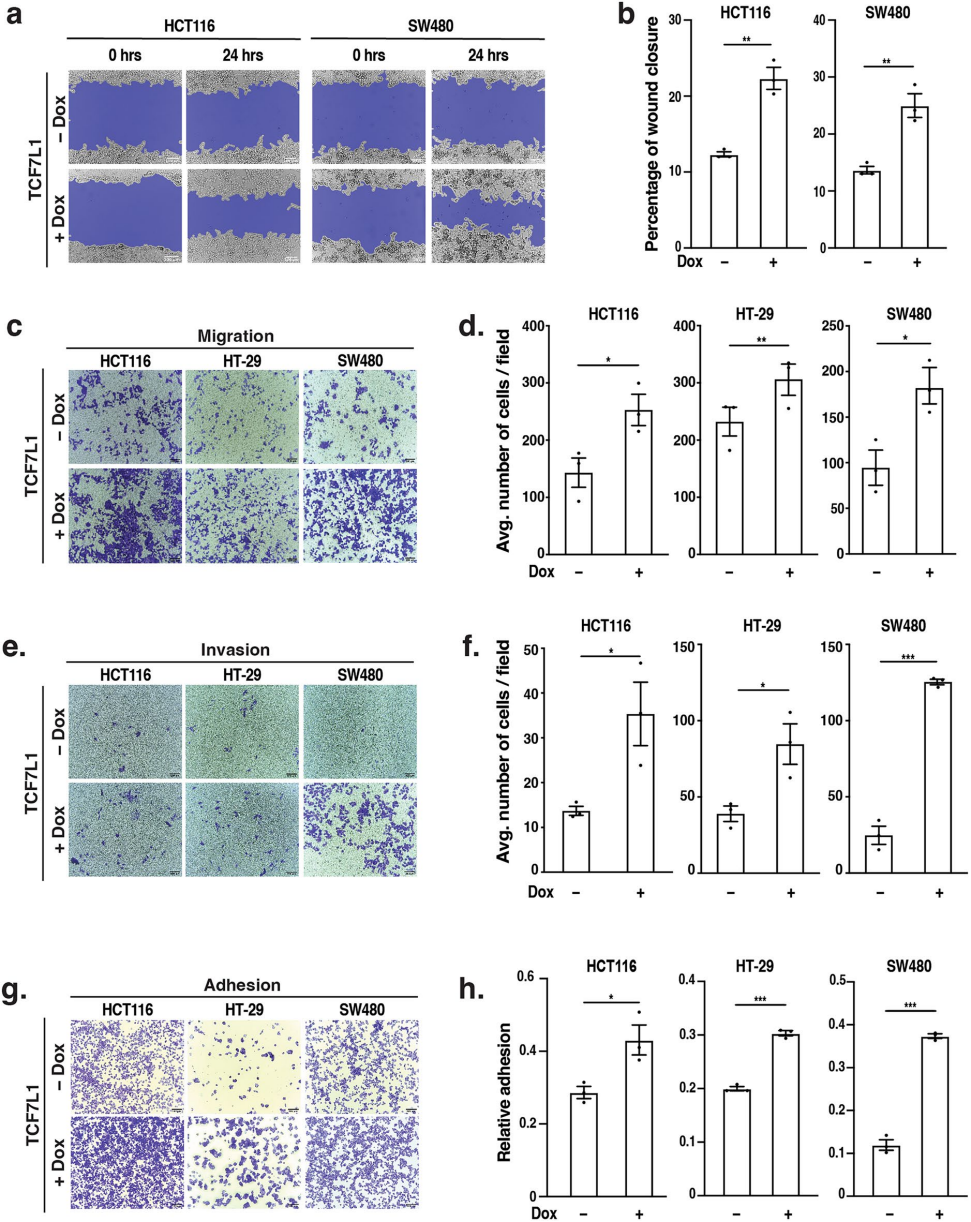
TCF7L1 promotes colorectal cancer cell migration, invasion and adhesion. (**a**) Representative images and (**b**) quantification of wound closure in stable HCT116 and SW480 treated without or with Dox to induce TCF7L1 expression. Scratch wounds were assessed at 0 and 24 h. (**c**) Representative images and (**d**) quantification of stable HCT116, HT-29 and SW480 cells treated with or without Dox and subjected to transwell migration assays. (**e**) Representative images and (**f**) quantification of stable HCT116, HT-29 and SW480 cells treated with or without Dox and subjected to transwell invasion assays. (**g**) Representative images and (**h**) quantification of stable HCT116, HT-29 and SW480 cells treated with or without Dox and subjected to cell adhesion assays. Scale bars are 100 µm. Data are represented as mean ± SEM (*p < 0.05, **p < 0.01, ***p < 0.001).

Given the enrichment for genes associated with EMT in our transcriptome analysis, we hypothesized that TCF7L1 regulates CRC cellular migration. To test this hypothesis, we utilized the stable HCT116 cell lines, established Dox-inducible *TCF7L1* and *TCF7L1*^MUT^ HT-29 and SW480 CRC cell lines (Supplementary Fig. S2) and subjected these cells to multiple independent assays. Dox-induced TCF7L1 expression promoted migration of HCT116 and SW480 cells in scratch-wound healing assays after 24 h (Fig. 2a,b). In transwell assays using cell inserts, we found that TCF7L1 promoted migration and invasion of HCT116, HT-29 and SW480 cells (Fig. 2c–f). TCF7L1 also increased adhesion of HCT116, HT-29 and SW480 cells to collagen I-coated plates (Fig. 2g,h). In contrast, we found that expression of TCF7L1^MUT^ had no effect in any of these assays (Supplementary Fig. S3).

**Figure 3:**
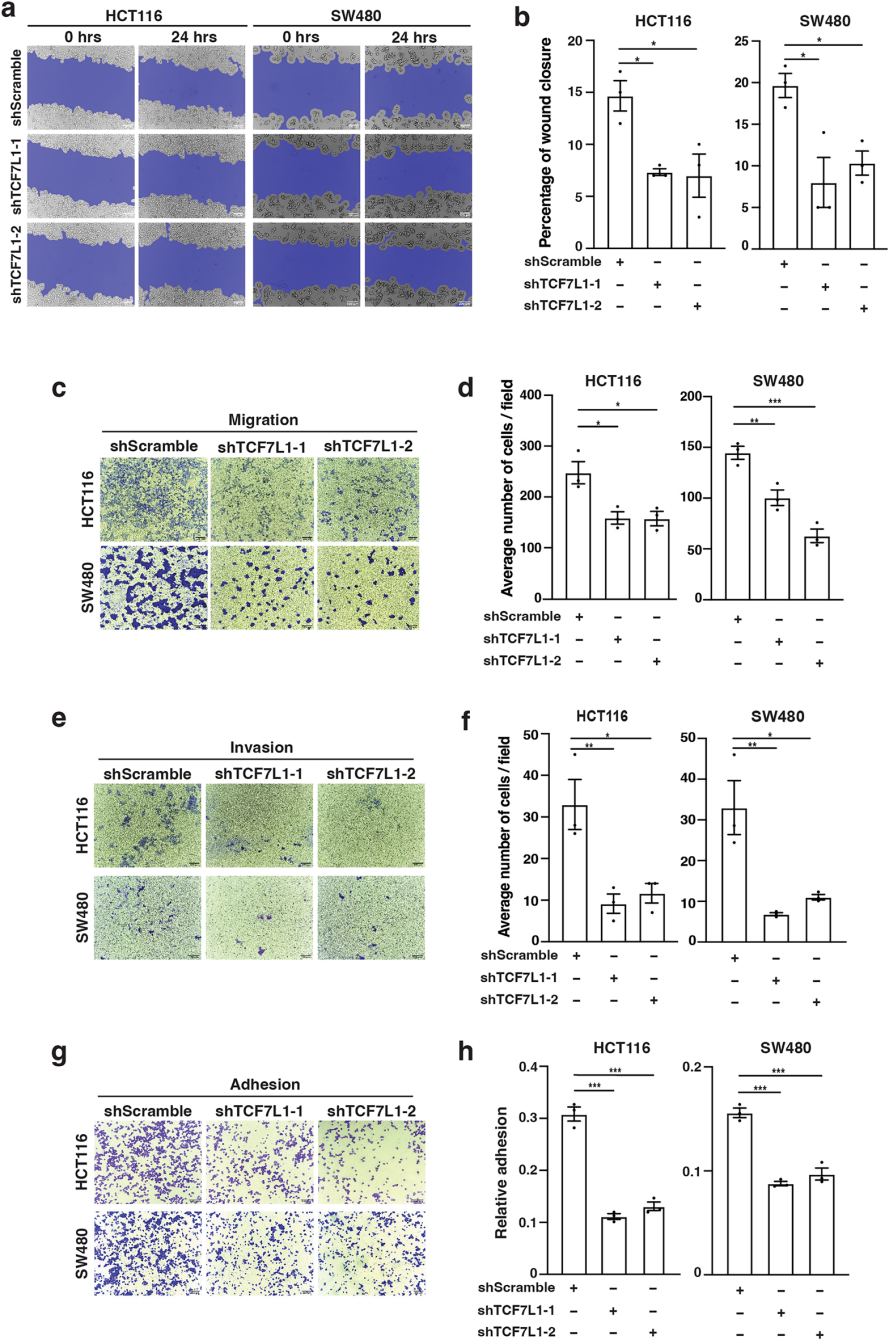
TCF7L1-depletion reduces colorectal cancer cell migration, invasion and adhesion. (**a**) Representative images and (**b**) quantification of wound closure in control (shScramble) and TCF7L1 knock-down HCT116 and SW480 cells. Scratch wounds were assessed at 0 and 24 h. (**c**) Representative images and (**d**) quantification of control (shScramble) and TCF7L1 knock-down HCT116 and SW480 cells subjected to transwell migration assays. (**e**) Representative images and (**f**) quantification of control and TCF7L1 knock-down HCT116 and SW480 cells subjected to transwell invasion assays. (**g**) Representative images and (**h**) quantification of control and TCF7L1 knock-down HCT116 and SW480 cells subjected to adhesion assays. Scale bars are 100 µm. Data are represented as mean ± SEM (*p < 0.05, **p < 0.01, ***p < 0.001).

As a complementary approach, we conducted the same assays in *TCF7L1*-depleted cells. In our previous studies, we identified two independent shRNAs that targeted distinct regions of the *TCF7L1* transcript^9,16^. Transduction of HCT116 and SW480 cells with lentiviruses harboring these shRNAs (designated shTCF7L1-1 and shTCF7L1-2) effectively reduced *TCF7L1* levels relative to a scramble control (Supplementary Fig. S4). Knocking down *TCF7L1* significantly impaired healing of scratch wounds, migration and invasion of HCT116 and SW480 cells (Fig. 3a–f). In addition, *TCF7L1* depletion reduced adhesion of these cells to collagen I-coated plates (Fig. 3g,h).

HCT116 and SW480 cells contain mutations in *CTNNB1* and *APC*, respectively, that contribute to activation of downstream Wnt*/*β-catenin signaling. To determine whether hyperactive Wnt signaling was required for *TCF7L1* migratory phenotypes, we used RKO cells which have wild-type *CTNNB1* and *APC* alleles. Depleting *TCF7L1* reduced cell migration and invasion of RKO cells indicating that a mutant Wnt signaling pathway is not required for TCF7L1-dependent regulation of these cellular processes (Supplementary Fig. S5).

### TCF7L1 represses *GAS1* gene expression

**Figure 4:**
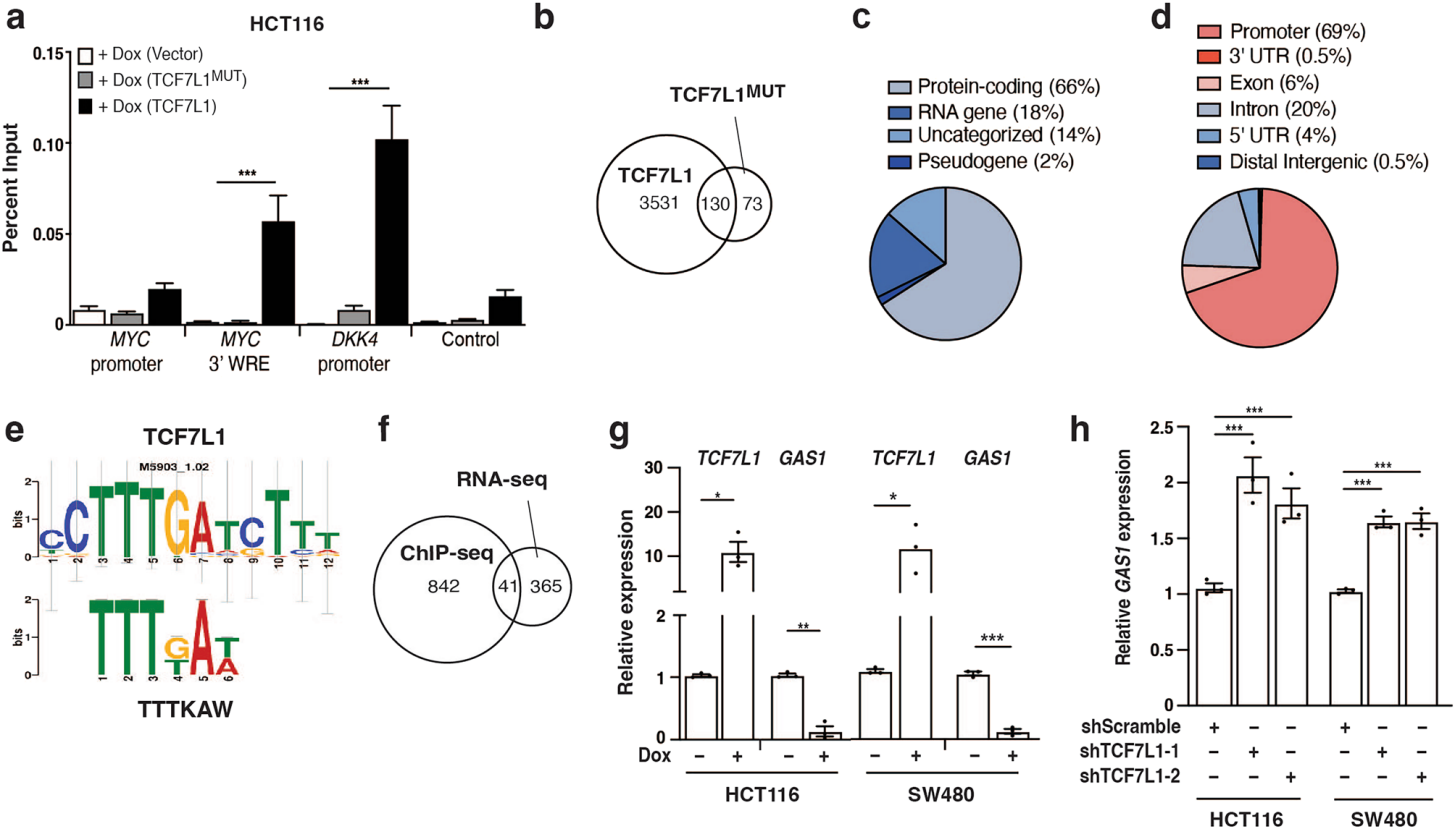
TCF7L1 represses *GAS1* gene expression. (**a**) ChIP-qPCR analysis of anti-FLAG immunoprecipitated and purified DNA in control (vector) and stable TCF7L1 and TCF7L1^MUT^ HCT116 cells treated with Dox. (**b**) Venn diagram of TCF7L1 and TCF7L1^MUT^ binding regions identified in anti-FLAG ChIP-seq experiments from the respective stable HCT116 cells treated with Dox. (**c**) Distribution of TCF7L1 binding regions relative to the nearest annotated gene. (**d**) Distribution of TCF7L1 binding regions within protein-coding genes. (**e**) Sequence logo for the TCF7L1 motif identified within TCF7L1 enriched regions (*p *value = 0.0067). (**f**) Venn diagram of TCF7L1-mediated downregulated genes and genes with TCF7L1 binding sites within 2.5 kb of the TSS of protein-coding genes. (**g**) RT-qPCR analysis of *TCF7L1* and *GAS1* transcript levels in control (−Dox) and Dox-treated stable HCT116 and SW480 cells. (**h**) RT-qPCR analysis of *GAS1* transcript levels in HCT116 and SW480 cells transduced with lentiviruses expressing the indicated shRNAs. In (**g**) and (**h**), relative gene expression data are normalized to *ACTB* levels*.* Data are represented as mean ± SEM (*p < 0.05, **p < 0.01, ***p < 0.001).

One limitation of RNA-seq analyses is that they cannot discern between direct and indirect effects in terms differential regulation of gene expression. To further define target genes whose expression are likely controlled by TCF7L1 binding to DNA regulatory elements, we conducted chromatin immunoprecipitation coupled with high-throughput sequencing (ChIP-seq). We performed these assays in *TCF7L1*-depleted HCT116 cells that re-expressed exogenous TCF7L1 or TCF7L1^MUT^ after Dox treatment. Prior to sequencing, we conducted ChIP-qPCR using primer sets that assessed previously characterized TCF7L1 binding sites at the *MYC* 3’ WRE and *DKK4* promoter, as well as the *MYC* promoter and a control site^9,16^. We detected little binding across all regions in Dox-treated cells expressing TCF7L1^MUT^ or an empty vector control (Fig. 4a). In Dox-treated cells expressing exogenous TCF7L1, we detected robust TCF7L1 binding to known binding sites at the *MYC* 3’ WRE and the *DKK4* promoter (Fig. 4a). Having validated the specificity of the ChIP, we next established libraries for next generation sequencing to identify TCF7L1 binding regions across the CRC genome.

After aligning the sequences to the human reference genome, we identified 3,661 TCF7L1 and 203 TCF7L1^MUT^ binding regions (Fig. 4b; Supplementary Table S4). Of the 3,531 unique TCF7L1 binding regions, most were proximal to protein-coding genes, and fewer localized near RNA genes, genes encoding uncategorized transcripts and pseudogenes (Fig. 4c). Further analysis of binding regions near protein-coding genes indicated that the majority (69%) mapped to gene promoters (Fig. 4d). We then identified enriched motifs within TCF7L1-bound promoter regions using Discriminative Regular Expression Motif Elicitation (DREME) and interrogated known transcription factor binding motifs using TOMTOM^29,30^. The TTTKAW motif associated with TCF7L1, TCF7L2, and LEF1 was amongst the top five enriched motifs (Fig. 4e, Supplementary Table S5). Notably, the TCF7L1 consensus binding motif was also enriched in these TCF7L1 binding regions (*p *value = 0.00067) (Fig. 4e). Together, these findings indicate that most TCF7L1 binding regions are located near (< 2.5 kb) or within protein-coding gene promoters and that it likely associates with its consensus DNA-binding motif at these sites.

To identify potential direct targets of TCF7L1-mediated repression, we overlapped the RNA-seq and ChIP-seq datasets. We considered the 397 genes whose expression were downregulated by TCF7L1 and the 883 protein-coding genes with TCF7L1 binding sites within 2.5 kb of the TSS. This conservative analysis identified 41 genes (Fig. 4f; Supplementary Table S6). While the defined Wnt/β-catenin target *LGR5* was among these genes^15^, we did not identify a more expansive Wnt target gene signature. Next, we conducted gene set overlap analysis to identify those genes repressed by TCF7L1 that may regulate cell migration and invasion. Of the 41 targets we identified, four were amongst the 200 genes associated with the MSigDB “Hallmarks of EMT” data set^27^. These four genes included frizzled class receptor 8 (*FZD8*), growth arrest specific 1 (*GAS1)*, laminin subunit alpha 3 (*LAMA3*), and tenascin C (*TNC*) (Supplementary Table S7). Amongst these, we focused on *GAS1* as it has been previously identified as a negative regulator of colorectal carcinogenesis and metastasis^23^. Induction of TCF7L1 reduced expression of *GAS1* transcripts in the stable HCT116 and SW480 cell lines (Fig. 4g). In addition, knocking down *TCF7L1* increased *GAS1* transcript levels in HCT116 and SW480 cells (Fig. 4h). Together, these data indicate that TCF7L1 negatively regulates *GAS1* expression in these CRC cell lines.

### *GAS1* suppresses the TCF7L1-mediated increase in colorectal cancer cell migration and invasion

**Figure 5:**
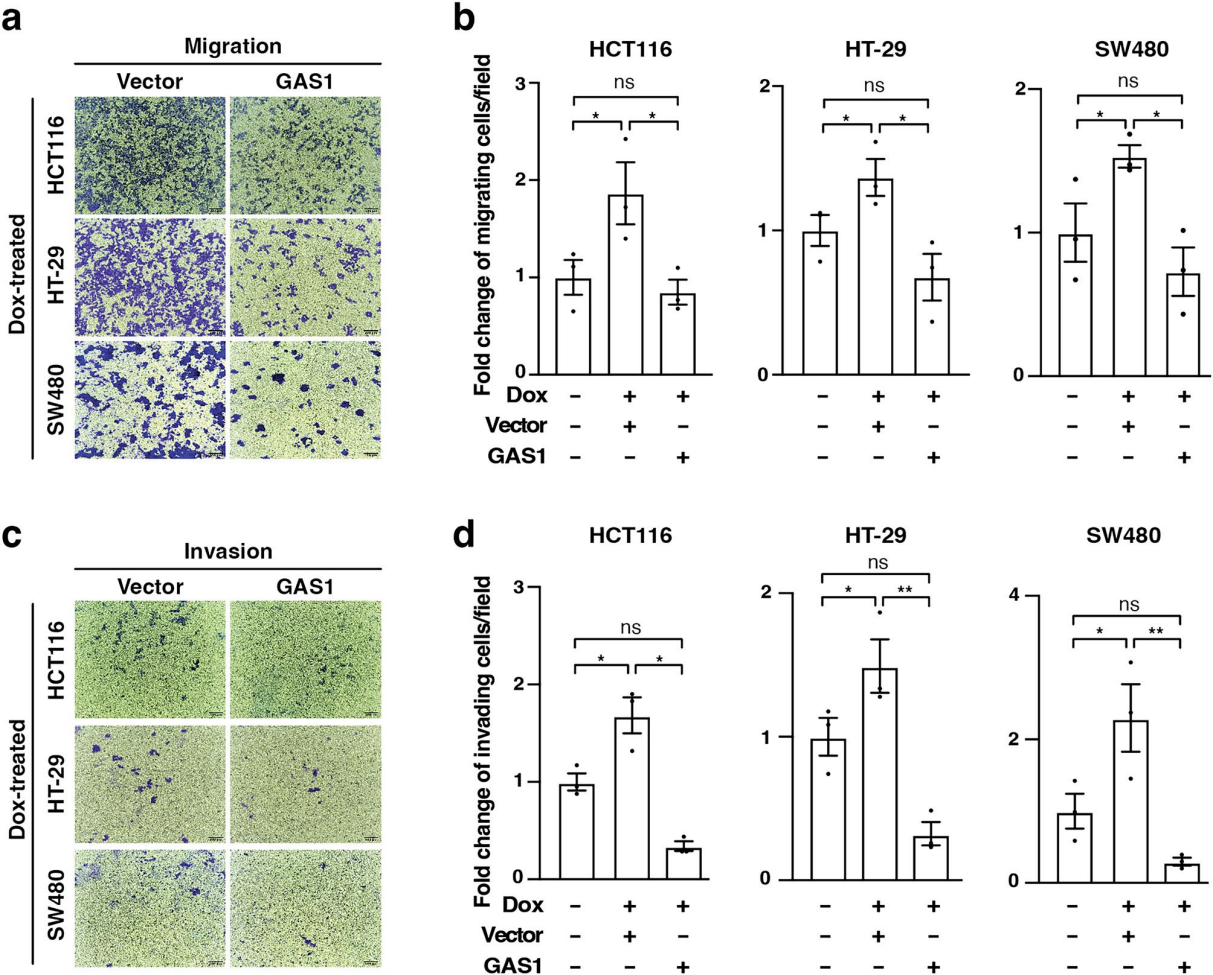
*GAS1* suppresses TCF7L1-mediated increase in colorectal cancer cell migration and invasion. (**a**) Representative images of the indicated Dox-treated CRC cell lines that were transfected with vector or a *GAS1* expression plasmid and subjected to transwell migration assays. (**b**) Quantification of migrated cells +/−Dox treatment that were transfected with the plasmids indicated. (**c**, **d**) as in (**a**, **b**) except cells were subjected to transwell invasion assays. Scale bars are 100 µm. Data are represented as mean ± SEM (*p < 0.05, **p < 0.01, ***p < 0.001).

We conducted rescue experiments to determine whether *GAS1* repression contributed to the increased migration and invasion phenotypes in TCF7L1-expressing CRC cells. First, we confirmed *GAS1* protein levels were increased following the transfection of a plasmid expressing *GAS1* cDNA in Dox-treated TCF7L1 stable HCT116 cells (Supplementary Fig. S6). As reported earlier (Fig. 2c–f), Dox-induced TCF7L1 promoted HCT116**,** HT-29, and SW480 cell migration (Fig. 5a,b). In parallel cultures, transfection of a plasmid containing *GAS1* cDNA in TCF7L1-expressing cells restored baseline levels of migration (Fig. 5a,b). These *GAS1*-dependent rescue phenotypes were also noted in cell invasion assays (Fig. 5c,d). In a complimentary approach, we found that knocking down *GAS1* rescued the reduced cellular migration phenotypes seen in *TCF7L1-*depleted HCT116, HT-29 and SW480 cells (Supplementary Fig. S7). Together, these results indicate that repression of *GAS1* expression by TCF7L1 contributes to CRC migratory potential.

## Discussion

Dysregulation of the Wnt/β-catenin signaling pathway occurs in the majority of sporadic CRCs as a result of activating mutations in key signaling components^2,4,5^. Considering that TCFs are critical regulators of Wnt/β-catenin target genes, studies have focused on elucidating the mechanism by which each factor contributes to CRC pathogenesis. Most reports focused on the roles TCF7L2, TCF7, and LEF1 in this cancer^7,31,32^. However, several reports have begun to define the role of TCF7L1 in CRC^9,13,15,16^. In this study, we confirm the role of TCF7L1 as a transcriptional repressor in CRC and identify novel targets of TCF7L1-mediated repression using comparative analysis of RNA- and ChIP-sequencing data sets. Furthermore, we highlight a novel role for TCF7L1 in promoting CRC cell migration, invasion, and adhesion, in part, through repression of *GAS1* gene expression.

Members of the TCF family of transcription factors have a highly-conserved HMG DNA-binding domain that facilitates their association to consensus TCF DNA-binding elements^7,8^. Earlier genome-wide TCF ChIP studies found that a substantial fraction of TCF-bound DNA fragments lacked a consensus TCF motif suggesting that it may be indirectly recruited to chromatin^33,34^. In line with this possibility, we found that TCF7L1^MUT^ did occupy a relatively small number genomic binding sites. However, we demonstrated that the DNA-binding capacity of TCF7L1 was required for its function as a transcriptional repressor and for TCF7L1-dependent promotion of CRC cellular migration. Therefore, repression of target genes through direct binding of TCF7L1 to WREs is likely the primary mechanism which accounts for its oncogenic function in CRC.

Despite their multiple conserved protein–protein interaction domains, the TCF family members differentially regulate Wnt target gene expression^7,8^. The heterogeneity in TCF member transcriptional activity provokes questions regarding what differentiates TCF function as transcriptional activators or repressors. Despite a strong similarity to other TCF members, TCF7L1 primarily functions as a transcriptional repressor in CRC^9,13,16^. Of the four family members, TCF7L1 has been shown to have the strongest binding to TLE corepressor proteins, which may partially explain its role as a transcriptional repressor in CRC^35^. Similarly, TCF7L1 is one of two TCF members that contain PXDLS motifs that modulate their association with corepressor CtBP^7,36^. Despite the known interaction of TCF7L1 with these corepressors, further work is needed to determine how these associations account for TCF7L1-mediated repression of target genes. Our identification of 41 TCF7L1-regulated target genes that also contain TCF7L1 promoter occupancy will enable us to address this question in future research.

Given the limited identification of target genes to date, we aimed to identify transcripts that were regulated by TCF7L1 in HCT116 CRC cells. In agreement with the role of TCF7L1 as a transcriptional repressor, the majority of differentially expressed genes were downregulated upon exogenous TCF7L1 expression. Notably, gene ontology (GO) and gene set enrichment analysis (GSEA) of the differentially expressed genes did not reveal a Wnt target signature. This finding is consistent with previous findings by our group and others that TCF7L1-regulated gene sets lacked a Wnt target signature^9,13^. We propose three possible explanations as to why TCF7L1-regulated gene sets include only a small subset of Wnt target genes. Firstly, given the constitutively active Wnt signaling in CRC cells, TCF binding sites at known Wnt target genes may be occupied by other TCF members. Secondly, it is plausible that the list of Wnt target genes is more expansive than what has been characterized to date. Thirdly, the lack of Wnt target gene signature may suggest that TCF7L1 may act as an oncogene independently of the Wnt/β-catenin signaling pathway in CRC.

In this study, GSEA analysis revealed enrichment of genes associated with EMT in control cells. Furthermore, we found that TCF7L1 promotes CRC cell migration, invasion, and adhesion in CRC cells, phenotypes highly prevalent in cells undergoing EMT. During EMT, cancer cells develop increased motility and invasiveness as they lose their traditional epithelial properties and gain mesenchymal cell characteristics^37^. The Wnt/β-catenin signaling pathway has been shown to induce EMT in CRC^38^. Because TCF7L1 is considered primarily a repressor of Wnt target genes, TCF7L1-mediated promotion of EMT-associated phenotypes was a surprising finding. While TCF7 and LEF1 have been implicated as positive regulators of migration and invasion in CRC cells, TCF7L2 has been shown to inhibit these phenotypes^39–42^. These results demonstrate the complexities associated with TCF-dependent regulation of EMT. Detailed experiments using tumor models in vivo are needed to further understand how TCF family members govern EMT and metastases.

Our functional genomics approach identified four genes that are associated with EMT: *FZD8, GAS1, LAMA3,* and *TNC.* Of these, *GAS1* has been previously implicated as a tumor suppressor in CRC^23^. Li et al. demonstrated that *GAS1* negatively regulated aerobic glycolysis by reducing expression of genes encoding key glycolytic enzymes such as *GLUT4*, *HK2* and *LDHB*^23^. In addition, *GAS1* inhibited CRC migration and invasion, in part, by promoting expression of E-cadherin and suppressing expression of vimentin, N-cadherin and Snail^23^ Furthermore, *GAS1* decreased activity of the AMPK/mTOR/p70S6K signaling cascade^23^. Thus by impacting cellular metabolism and key signaling pathways, *GAS1* was shown to inhibit early processes in EMT^23^. In our study, *GAS1* expression was significantly downregulated in CRC cells expressing exogenous TCF7L1 and restoration of *GAS1* expression suppressed the TCF7L1-mediated increase of CRC cell migration and invasion in vitro*.* Together, these data indicate that TCF7L1 promotes CRC migration and invasion, in part, by repressing metastatic inhibitor *GAS1.* Additional work is needed to determine whether TCF7L1-dependent regulation of *FZD8*, *LAMA3* and *TNC* contributes to CRC migratory properties.

Our study implicates a novel role for TCF7L1 in regulation of CRC cell migration invasion and adhesion, phenotypes strongly associated with EMT and metastasis. As a regulator of target genes associated with EMT, strategies targeting TCF7L1, its down-regulated targets, or its corepressors may offer a potential therapeutic strategy to reduce metastasis in CRC patients.

## Methods

### Cell culture

Human colorectal cancer cell lines HCT116, HT-29, SW480, and RKO were purchased from American Type Culture Collection (ATCC). HEK293/FT cells were purchased from Invitrogen. RKO cells were grown in Eagle’s Minimal Essential Medium (EMEM) and all others were grown in Dulbecco’s Modified Eagle’s Medium (DMEM, Corning). The growth media was supplemented with 10% fetal bovine serum (FBS, Gemini Bio), 2 mM Glutamax (Gibco), and 1% penicillin/streptomycin (Gibco). The cells were cultured at 37 °C in 5% CO_2_. HEK293FT cells were maintained in media containing 500 g/ml G418 (VWR).

### Plasmids

pCMV6-entry-TCF7L1 and pCMV6-*GAS1* were purchased from Origene, pCW57.1-empty vector was purchased from Addgene, and pCMV-Tag2B-empty vector was purchased from Stratagene. The pCMV6-entry-TCF7L1 plasmid expressing FLAG-epitope tagged TCF7L1 cDNA was engineered to contain silent mutations rendering the cDNA resistant to shRNAs using the QuikChange Lightning Site-Directed Mutagenesis kit (Agilent Technologies)^15^. TCF7L1 cDNA harboring the shRNA-resistant mutations was amplified using oligonucleotides that incorporate EcoRI/HindIII restriction sites by PCR and the resultant fragment was inserted in the pCMV-Tag2B-empty vector to generate pCMV-Tag2B-TCF7L1. Site-directed mutagenesis was used to generate nucleotide substitutions within the HMG DNA-binding domain that resulted in a lysine to proline substitution at residue 387 and a proline to lysine substitution at residue 411 to generate pCMV-Tag2B-TCF7L1^MUT^. TCF7L1 cDNA sequences were confirmed using Sanger sequencing. To generate doxycycline (Dox)-inducible plasmids, TCF7L1 and TCF7L1^MUT^ cDNAs were amplified using oligonucleotides that incorporate NheI/AgeI restriction sites by PCR and the resultant fragments were inserted in the pCW57.1-empty vector to generate pCW57.1-TCF7L1 and pCW57.1-TCF7L1^MUT^. Oligonucleotide sequences used for PCR are listed in Supplementary Table S8.

### Luciferase reporter assays

Luciferase reporter assays were conducted as previously described^15^. Briefly, HCT116, HT-29, or SW480 cells were seeded in quadruplicate in 24-well plates and transfected the following day with Lipofectamine 2000 (Invitrogen). Each reaction contained 100 ng of the luciferase reporter plasmid TOPflash and 2 ng of the transfection control pLRL-SV480 *Renilla*. Total concentration of DNA was adjusted to 500 ng per reaction using pBlueScript II SK (-). Where indicated, transfection reactions contained 100 ng of pCMV-Tag2B-empty vector, pCMV-Tag2B-TCF7L1 or pCMV-Tag2B-TCF7L1^MUT^. Transfection media was replaced after 6 h and cells were lysed in passive lysis buffer (Biotium) after 24 h. Luciferase levels were measured using the dual luciferase single tube assay kit (Biotium) on a Glomax 20/20 single chamber luminometer (Promega). Data are presented as relative luciferase levels (firefly luciferase/*Renilla* luciferase).

### Stable cell lines

Stable cell lines were generated as previously described^15^. Briefly, HEK293FT cells were transfected with 3 µg of lentiviral packaging plasmids (pLP1, pLP2, and pLP/VSVG, Invitrogen) and 3 µg of pCW57.1-empty vector, pCW57.1-TCF7L1 or pCW57.1-TCF7L1^MUT^ using Lipofectamine 2000. Following transfection, media containing lentivirus were collected at 24- and 48-h and added to HCT116, HT-29, and SW480 cells for 8 h. Lentiviral infected HCT116, HT-29, and SW480 cells were selected using media supplemented with 1 µg/ml (HCT116) or 1.5 µg/ml (HT-29 and SW480) puromycin for two weeks. After selection, stable cell lines were maintained in 0.5 µg/ml puromycin. TCF7L1 or TCF7L1^MUT^ expression was induced by treating cell lines with media supplemented with doxycycline (Dox, Thermo Fisher) at 1 µg/ml for 18 h.

### Western blot analysis

Protein extracts from whole cell lysates were isolated in radioimmunoprecipitation assay (RIPA) buffer, quantified, and analyzed by western blot as previously described^43^. Blots were probed with primary antibodies against FLAG (Sigma-Aldrich, 1:1000 dilution), ɑ-tubulin (Sigma-Aldrich, 1:1000 dilution), *GAS1* (Abcam, 1:500 dilution) or β-actin (Cell Signaling Technology, 1:1000 dilution). After treatment with ECL reagents, blots were exposed to film and developed.

### Reverse transcription and quantitative real time PCR (RT-qPCR)

RNA was isolated using TRIzol reagent and cDNA was synthesized using Verso cDNA synthesis kit (Thermo-Fisher) following manufacturer guidelines. *TCF7L1*, *GAS1, ACTB* and *GAPDH* expression was measured using previously described methods^44^. Data is presented as relative expression using the 2^−ΔΔCT^ method after normalization to the *ACTB* or *GAPDH* housekeeping genes. Primer sequences are listed in Supplementary Table S8.

### Lentiviral shRNA-mediated knockdowns

Lentiviral plasmids (pLKO.1) encoding two independent *TCF7L1* shRNAs and a scramble shRNA control were obtained from Open Biosystems as previously described^9^. A lentiviral plasmid encoding *GAS1* shRNA was obtained from The Pennsylvania State College of Medicine’s Genome Sciences (RRID:SCR_021123) core facility. Sequences and clone numbers for shRNAs targeting *TCF7L1* are listed in Supplementary Table S8. To generate lentiviruses, HEK293FT cells were transfected with 3 µg lentiviral packaging plasmids (pLP1, pLP2, and pLP/VSVG) and 3 µg of the respective lentiviral shRNA plasmid construct using Lipofectamine 2000. Following transfection, media containing lentivirus was collected at 24- and 48-h, supplemented with 6 µg/ml hexadimethrine bromide and added to HCT116, HT-29, SW480 and RKO cells for 8 h. Cells were harvested for assays after 72 h.

### RNA-sequencing and analysis

RNA was isolated using TRIzol reagent from TCF7L1-depleted and Dox-treated HCT116 stable cells harboring plasmids encoding an empty vector control, TCF7L1, or TCF7L1^MUT^. Preparation of the cDNA libraries, transcriptome sequencing, and bioinformatic analyses were conducted by Novogene Co., Ltd. Briefly, an average of 27 million processed reads per sample were mapped to the reference human genome (GRCh38 /hg38) using STAR (v2.5) and aligned reads were counted using HTSeq (v0.6.1). Samples contained an average of 92% uniquely mapped reads and no GC content bias was detected. Quality assessment of the RNA-sequencing libraries is listed in Supplementary Table S9. Read counts were adjusted by edgeR program package through one scaling normalized factor. Differential expression analysis was performed using the edgeR R package (v3.16.5). *p* values were adjusted using the Benjamini and Hochberg method. Genes with adjusted *p* values < 0.05 and |log2foldchange|> 2 were considered differentially expressed and are listed in Supplementary Table S1. Gene ontology (GO) network analysis was conducted on differentially expressed genes using GOnet^45^. Gene set enrichment analysis (GSEA, v4.1.0) for The Molecular Signatures Database (MSigDB) hallmark gene set was performed with 1000 gene set permutations on normalized counts following software guidelines^27,28^.

### Scratch-wound assay

Control, Dox-treated, or TCF7L1-depleted cells (1 × 10^6^) were seeded in triplicate in 12-well plates and cultured to approximately 100% confluency. A vertical scratch was applied to the monolayer using a 10 µl plastic pipette tip. Non-adherent cells were removed by gentle washes with 1× PBS and cells were cultured in serum-free media. Wound areas were imaged using an inverted, brightfield Revolve microscope (Echo) at 0 h and 24 h and quantified using an automated wound healing ImageJ plugin^46^. Data are presented as percentage of wound closure at 24 h.

### Transwell migration and invasion assay

Transwell migration and invasion assays were performed in 24-well plates with uncoated or Matrigel-coated polycarbonate membranes containing 8.0 µm pores, respectively. Stable TCF7L1 and TCF7L1^MUT^ HCT116, HT-29, or SW480 cells were seeded (1 × 10^4^ cells per well) in serum-free media, + /- Dox, in the upper chambers of the transwell inserts in triplicate. For rescue experiments, Dox-treated HCT116, HT-29, or SW480 cells expressing FLAG-TCF7L1 were transfected with pCMV-Tag2B empty vector or pCMV6-*GAS1* using Lipofectamine LTX reagent according to manufacturer guidelines prior to seeding. Media containing 10% FBS was added to the lower chambers. Chambers were incubated at 37 °C, 5% CO_2_ for 48 h to allow for cell migration or invasion. Cells that successfully migrated or invaded to the bottom of the membrane were fixed in 70% ethanol and then stained with 0.2% crystal violet. The total number of cells of four random fields of view were counted using standard color thresholding built-in to ImageJ software and the average was calculated. Stained cells were imaged using an inverted, brightfield Revolve microscope (Echo).

### Adhesion assay

Control and Dox-treated, stable TCF7L1 and TCF7L1^MUT^ cells were seeded (1 × 10^3^ cells per well) in serum-free media in 96-well plates pre-coated with 40 µg/ml collagen I (Sigma-Aldrich) in triplicate. Plates were pre-washed and blocked with PBS supplemented with bovine serum albumin. Cells were incubated on collagen-coated plates for 1 h at 37 °C, 5% CO_2_. Non-adhered cells were removed by gentle washing with 1× PBS and adherent cells were fixed in 4% paraformaldehyde solution and stained with 0.1% crystal violet. Crystal violet stain was eluted in 1% sodium dodecyl sulfate (SDS) and absorbance was measured using spectrophotometry to assess relative adhesion levels. Representative fields of view were imaged using an inverted, brightfield Revolve microscope (Echo). Data are presented as relative adhesion or absorbance at 570 nm.

### Chromatin immunoprecipitation (ChIP)

ChIP assays were conducted using the ChIP-IT High Sensitivity Kit (Active Motif) on stable HCT116 cells containing a vector control, TCF7L1, or TCF7L1^MUT^ and treated with Dox. Cross-linked and sheared chromatin was precipitated with 4 µg of anti-FLAG antibodies (Sigma-Aldrich) overnight at 4 °C on a rocking platform. Precipitated and purified DNA was amplified using qPCR in triplicate with primers listed in Supplementary Table S8 and data are presented as percent input. For ChIP-sequencing, prepared samples were sent to Novogene Co., Ltd. for library construction, sequencing, and bioinformatic analysis. Briefly, raw reads were trimmed using skewer software (v0.1.126), quality control was assessed using FastQC, clean reads were mapped against the human genome (GRCh38 /hg38) using BWA (v0.7.12), peaks were called for aligned reads using MACS2 (v2.1.0), and peaks were annotated using PeakAnnotator_Cpp (v1.4) or ChIPseeker package in R. Annotations were based on the nearest gene and genomic region where the peak was located. Motif discovery analysis was conducted using Discriminative Regular Expression Motif Elicitation (DREME)^29^ and quantified using TOMTOM^30^. Quality assessment of the ChIP-sequencing libraries is listed in Supplementary Table S10.

### Gene set overlap analysis

Overlap between the MSigDB “Hallmarks of Epithelial Mesenchymal Transition” gene set^27^ and TCF7L1-specific downregulated differentially expressed genes were computed using University of California San Diego and The Broad Institute’s “Investigate Human Gene Sets” feature at https://www.gsea-msigdb.org/gsea/msigdb/human/annotate.jsp.

### Statistical analyses

The data are presented as the mean ± the standard error of the mean (SEM). Each experiment was repeated at least three times. Data points are representative of three independent biological replicates with each containing at least three technical replicates. Statistical significance was calculated using a Student’s t-test, Welch’s t-test, or a one-way ANOVA followed by a Tukey’s or Dunnett’s test for multiple comparisons when appropriate. A *p* value < 0.05 was considered statistically significant and indicated as follows: *, *p* < 0.05; **, *p* < 0.01; ***, *p* < 0.001.

### Supplementary Information

Supplementary Information 1.

Supplementary Table S1.

Supplementary Table S2.

Supplementary Table S4.

Supplementary Table S6.

## Data Availability

The data sets generated during the current study are available in the Gene Expression Omnibus (GEO) repository (GSE241028; RNA-seq data, GSE241025; ChIP-seq data, GSE241026). All other data are available within this manuscript and its supplementary files. Requests for additional information should be addressed to the corresponding author.
