# Supplementary material for: TCF7L1 regulates colorectal cancer cell migration by repressing GAS1 expression
Source: Sci Rep. 2024 May 30;14:12477. doi: 10.1038/s41598-024-63346-8 (PMC11139868; doi:10.1038/s41598-024-63346-8)
Supplement: Supplementary file 1 — Supplementary Information 1. [file 41598_2024_63346_MOESM1_ESM.pdf]

Supplementary Material for:

TCF7L1 regulates colorectal cancer cell migration by repressing *GAS1* expression

Carli M. King, Wei Ding, Melanie A. Eshelman, and Gregory S. Yochum

This file contains:

Supplementary Figures S1- S8

Supplementary Tables S3, S5, S7, S8, S9, S10

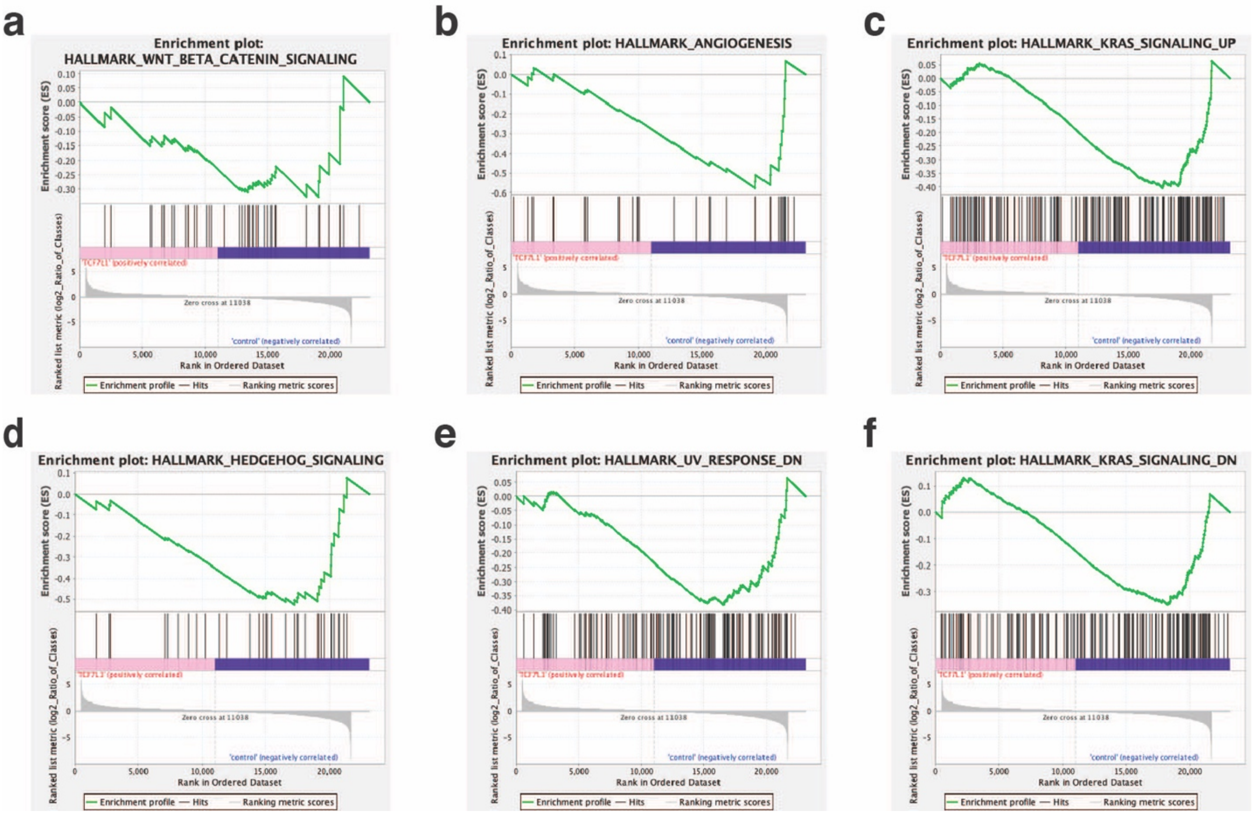

Suppl. Fig. S1. Gene set enrichment analysis (GSEA) of hallmarks of cancer gene sets. Differentially expressed genes from RNA-seq conducted in stable TCF7L1 HCT116 cells +/- Dox treatment were subjected to GSEA. (**a**) GSEA of genes comprising the hallmark Wnt/β-catenin target set in Dox-treated cells compared to control cells (-Dox) (normalized enrichment score = −1.06, nominal *p*-value = .38). (**b-f**) As in (**a**) except GSEA of genes comprising hallmarks of (**b**) angiogenesis, (**c**) upregulated KRAS signaling, (**d**) hedgehog signaling, (**e**) downregulated UV response, and (**f**) downregulated KRAS signaling sets were analyzed.

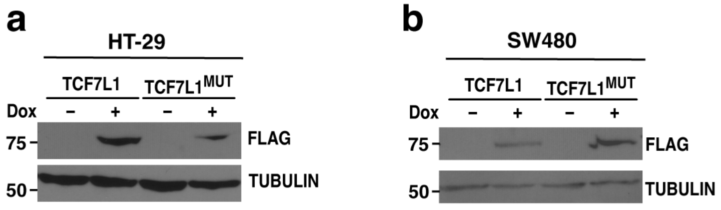

Suppl. Fig. S2. Dox-inducible colorectal cancer cell lines expressing TCF7L1 or TCF7L1^{MUT}. (**a**) Western blot analysis of TCF7L1 and TCF7L1^{MUT} in control (− Dox) or Dox-treated HT-29 and (**b**) SW480 cells.

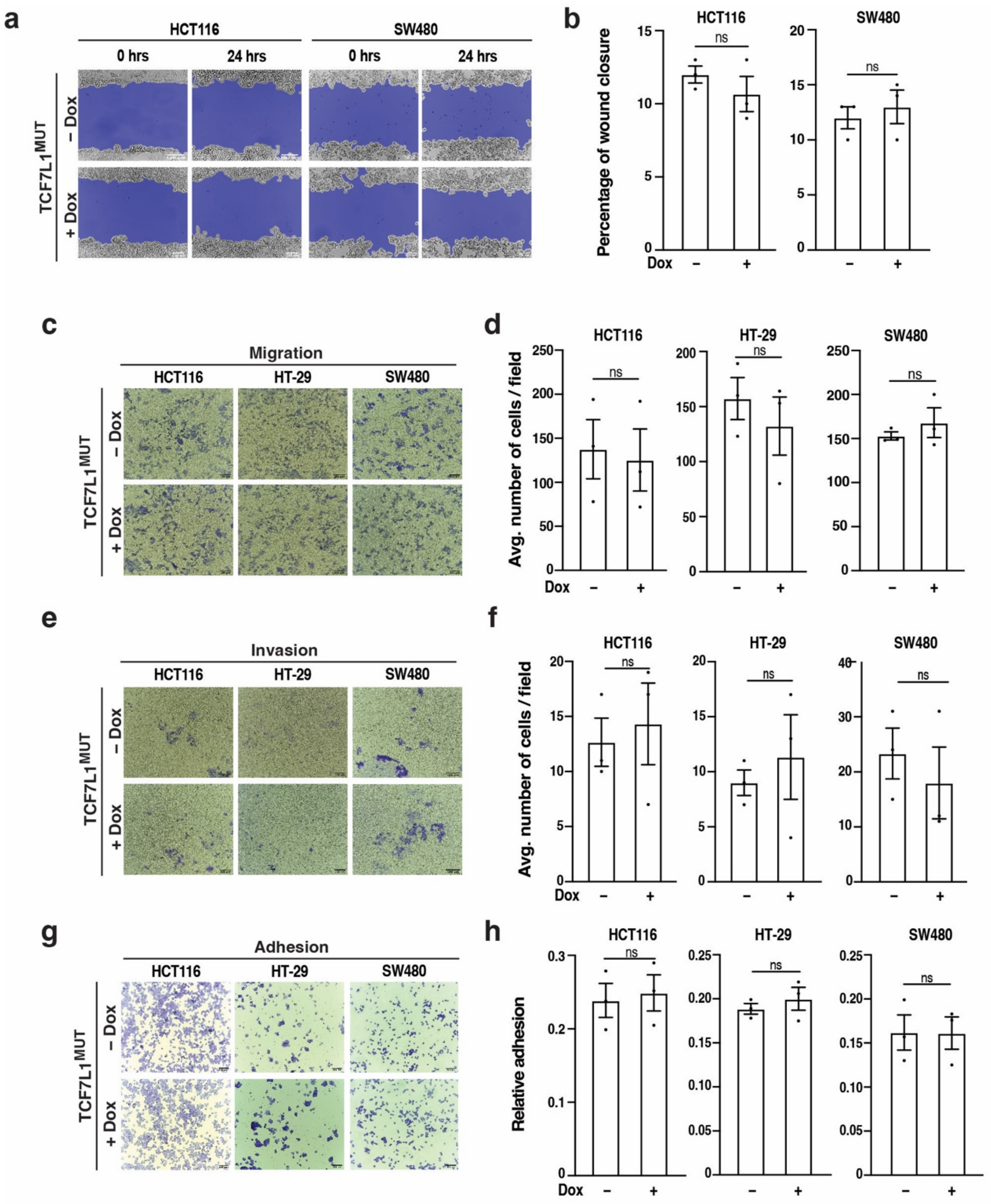

Suppl. Fig. S3. DNA-binding capacity is required for TCF7L1-mediated promotion of migration, invasion, and adhesion in colorectal cancer cells. (**a**) Representative images of control (-Dox) and Dox-treated stable TCF7L1^{MUT} HCT116 and SW480 cells subjected to scratch-wound migration assays. (**b**) Percentage of wound closure after 24 hours. (**c**) Representative images of control (-Dox) and Dox-treated stable TCF7L1^{MUT} HCT116, HT-29 and SW480 cells subjected to transwell migration assays. (**d**) Quantification of migrating cells per field of view. (**e**,**f**) as in (**b**,**c**) except cells were subjected to transwell invasion assays. (**g**,**h**) as in (**b**,**c**) except cells were subjected to cell adhesion assays. Scale bars are 100 µm. Error bars are mean ± SEM (ns, not significant).

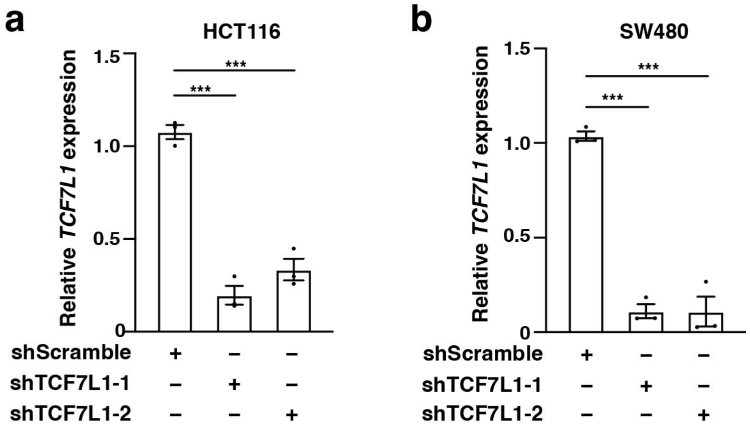

Suppl. Fig. S4. Two independent shRNAs deplete TCF7L1 transcripts in HCT116 and SW480 cell lines. RT-qPCR analysis of (**a**) HCT116 or (**b**) SW480 cells transduced with lentiviruses expressing either a scrambled shRNA sequence (shScramble) or two independent shRNAs targeting *TCF7L1* (shTCF7L1-1 and shTCF7L1-2). Relative expression values are normalized to *ACTB*. Error bars are mean ± SEM (*** $p < 0.001$).

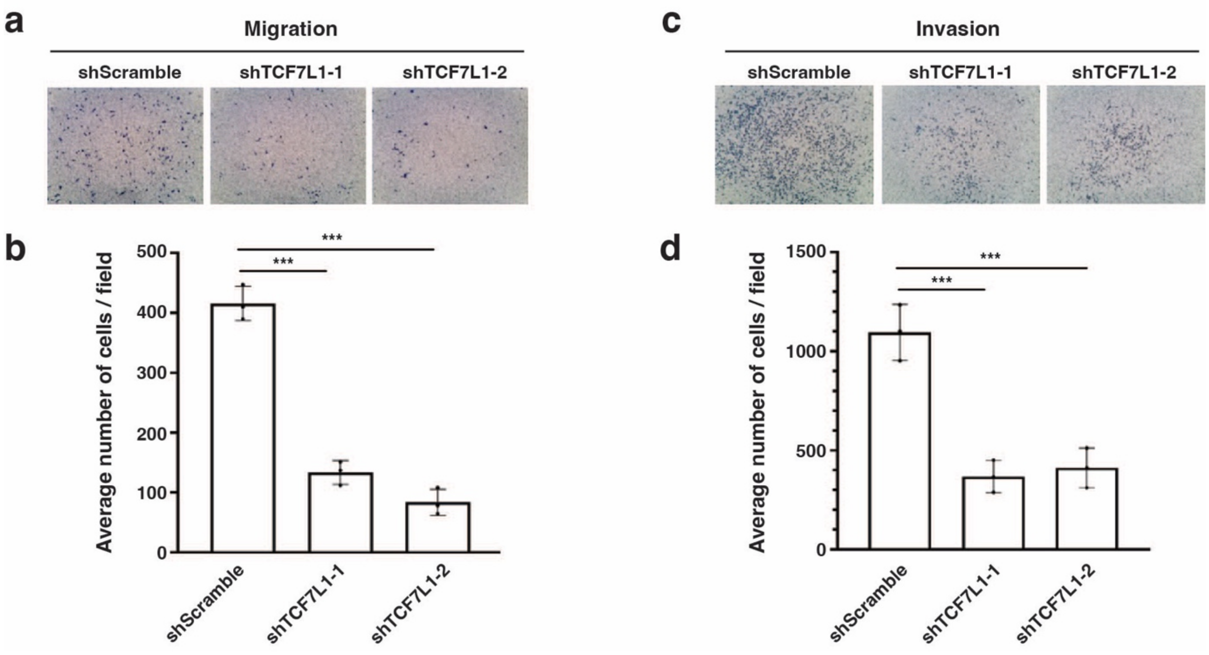

Suppl. Fig. S5. TCF7L1 depletion reduces RKO cell migration and invasion. (**a**) Representative images and (**b**) quantification of control (shScramble) and TCF7L1 knock-down RKO cells subjected to transwell migration assays. (**c**) Representative images and (**d**) quantification of control (shScramble) and TCF7L1 knock-down RKO cells subjected to transwell invasion assays. Error bars are mean ± SEM (*** $p < 0.001$).

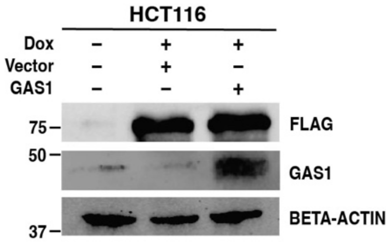

Suppl. Fig. S6. Analysis of GAS1 expression in stable TCF7L1 HCT116 cells. Western blot analysis of FLAG-TCF7L1 and GAS1 in control (-Dox) and Dox-treated stable HCT116 cells that were transfected with an empty vector or a plasmid encoding *GAS1*.

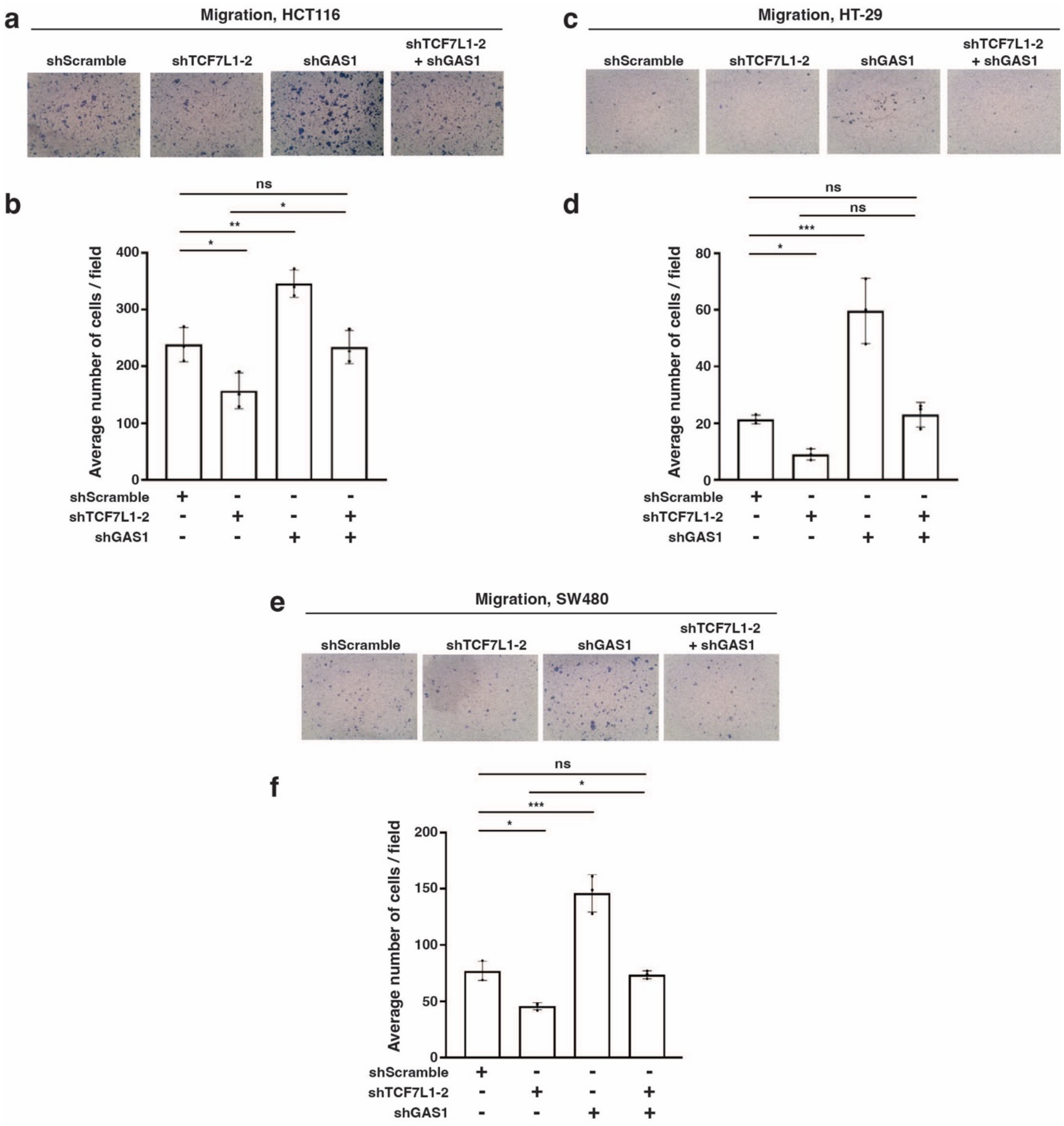

Suppl. Fig. S7. GAS1 is required for reduced migration in TCF7L1-depleted CRC cells. (**a**) Representative images and (**b**) quantification of control (shScramble), TCF7L1 knock-down, GAS1 knock-down, and TCF7L1 + GAS1 knock-down HCT116 cells subjected to transwell migration assays. (**c**, **d**) and (**e**, **f**) as in (**a**, **b**) except migration assays were conducted in HT-29 and SW480 cells, respectively. Error bars are mean ± SEM (* $p < 0.05$; ** $p < 0.01$; *** $p < 0.001$; ns = not significant).

Suppl. Fig. S8. Full blots

Related to Figure 1c:

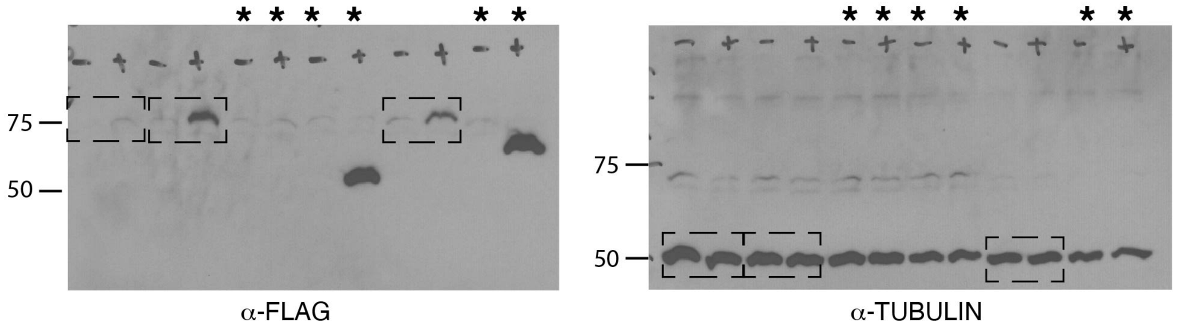

* Extracts from other stable HCT116 cells expressing mutant forms of TCF7L1 that were not evaluated in the current study.

Related to Figure S2a:

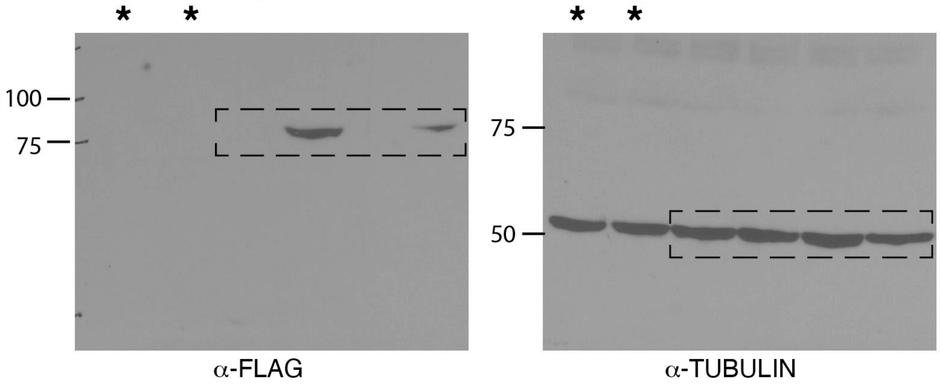

* Lanes 1 and 2 are from RKO extracts which were used as negative controls

Related to Figure S2b:

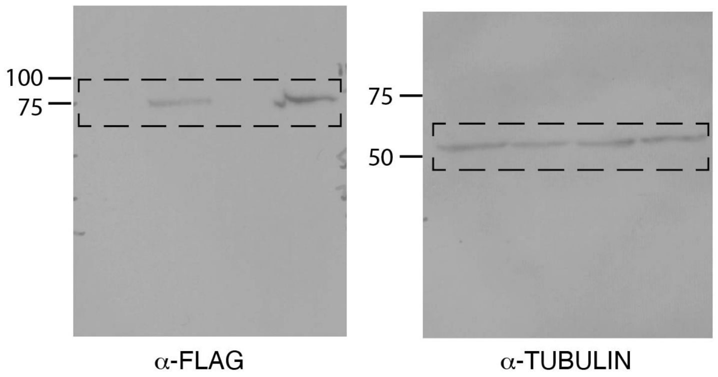

Suppl. Fig. S8. Full blots-continued

Related to Figure S6:

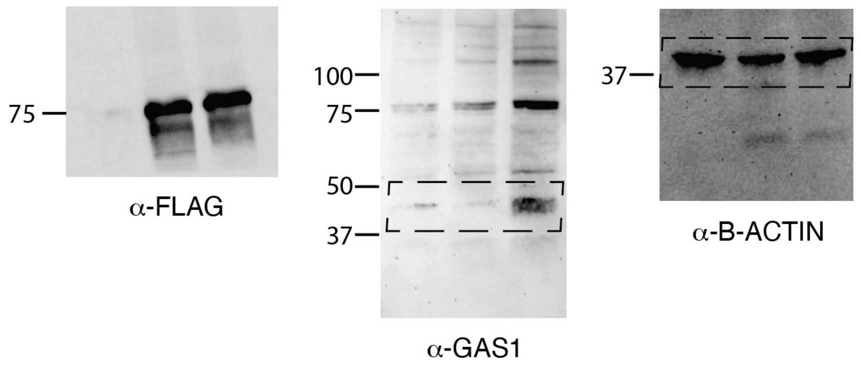

Supplementary Table S3. Gene set enrichment analysis of TCF7L1 DEGs

Gene Set	Normalized Enrichment Score	Nominal *p*-value
HALLMARK_EPITHELIAL_MESENCHYMAL_TRANSITION	− 1.78	0.000
HALLMARK_ANGIOGENESIS	− 1.76	0.003
HALLMARK_KRAS_SIGNALING_UP	− 1.65	0.001
HALLMARK_HEDGEHOG_SIGNALING	− 1.65	0.006
HALLMARK_UV_RESPONSE_DOWN	− 1.51	0.011
HALLMARK_KRAS_SIGNALING_DOWN	− 1.39	0.025

Supplementary Table S5. Top five motifs enriched in TCF7L1 binding regions

Enriched motif	Associated transcription factors	*p*-value
RTAAAY	FOXL1	2.62008e-05
	FOXD4L4	0.000101548
	HOXA9	0.000355372
	FOXG1	0.000359097
	FOXO4	0.000556732
	FOXF1	0.000613028
	FOXD2	0.000658128
	FOXJ2	0.000803323
	FOXK1	0.00105252
GGGWGGR	ZNF148	3.85918e-05
	MAZ	9.73016e-05
	KLF5	0.000245621
	SP1	0.000681601
	ZBTB7B	0.00111681
	KLF4	0.00133683
TTATCW	GATA1	1.0027e-05
	GATA2	1.50404e-05
	GATA6	1.70583e-05
	GATA4	1.81541e-05
	MECOM	0.000352069
	GATA5	0.000384069
ACCACWGC	GFI1	0.000516778
TTTKAW	TCF7L1	0.000667037
	TCF7L2	0.000236242
	LEF1	0.000238608

Supplementary Table S7. TCF7L1 target genes associated with EMT

Table 1. EMT hallmark genes downregulated in Dox-treated TCF7L1-expressing HCT116 cells

Gene Symbol	RNA-sequencing		ChIP-sequencing	
	Log2FoldChange	Adj. *p*-value	Log2FoldChange	Distance from TSS
FZD8	− 2.23	1.03E-04	2.37	− 1.146 kb
GAS1	− 2.46	1.41E-05	2.92	− 408 bp
LAMA3	− 2.54	5.54E-06	1.95	− 164 bp
TNC	− 2.31	2.73E-05	3.74	− 401 bp

Supplementary Table S8. List of oligonucleotide sequences used in this study

**Plasmid construction**		
Site-directed mutagenesis (shRNA resistance)	Forward	CTG AAC GAC AGG AAC TTT ATT GGA TAA ATG TGC TGG AGA TGG TGA CCT C
	Reverse	GAG GTC ACC ATC TCC AGC ACA TTT ATC CAA TAA AGT TCC TGT CGT TCA G
pCMV-Tag2b-TCF7L1	Forward	CCG AAT TCC CCC AGC TCG GCG
	Reverse	CCC AAG CTT TTA GTG GGC AGA CTT GGT GAC C
pCMV-Tag2b-TCF7L1^{MUT} (site directed mutagenesis)	Forward	CCG GAA GGA GCG GCA GCT TCA CTC GCA GCT CTA CCT AAC CTG GTC AGC CCG GGA
	Reverse	GCC AGC TCG TAG TAC TTG GCC TGT TCT TCT CGA GAC GGG TTG TGC CAC TTT CTT TCT TCC AAG G
pCW57.1 backbone plasmids	Forward	CTA GCT AGC GCC ACC ATG GAT TAC AAG G
	Reverse	TAG ACC GGT GTC GAC GGT ATC GAT AAG C
**RT-qPCR**		
TCF7L1	Forward	GCC ACT CCC TCT GCA GCT TTG G
	Reverse	TTT CTG GTT TGG TGG TGA GGG AGA
ACTB	Forward	GAG CAT CCC CCA AAG TTC ACA ATG
	Reverse	TGG CTT TTA GGA TGG CAA GGG ACT
GAS1	Forward	TCC AGA AAC TCC CAA CTC GTC TGC
	Reverse	AAT TGC TAA GGC CCC ACT GGT CAG
GAPDH	Forward	CCA GCA AGA GCA CAA GAG GAA GAG
	Reverse	CAA GGG GTC TAC ATG GCA ACT GTG
**shRNA-mediated knockdowns**		
shTCF7L1-1	Sequence	CCG GCG GGA CAA CTA TGG TAA GAA ACT CGA G**TT TCT TAC CAT AGT TGT CCC G**TT TTT G
	Clone number	TRCN0000021704
shTCF7L1-2	Sequence	CCG GCC AGC ACA CTT GTC TAA TAA ACT CGA G**TT TAT TAG ACA AGT GTG CTG G**TT TTT G
	Clone number	TRCN0000021705
shGAS1-3	Sequence	CCG GCC GCA CCG TCA TTG AGG ACA TCT CGA G**AT GTC CTC AAT GAC GGT GCG G**TT TTT G
shGAS1-3	Clone number	TRCN0000118139
**Chromatin immunoprecipitation (ChIP)**		
MYC promoter	Forward	TTT TGA CTG TGG CCC TGG ACT TTG
	Reverse	ACG CTG GAA GAT TTC TGG AGC TTG
MYC 3’ WRE	Forward	GCT CAG TCT TTG CCC CTT TGT GG
	Reverse	TAA CAC CTT CCC GAT TCC CAA GTG
DKK4 promoter	Forward	CAA AGG ATT ACT GAA GGT AGG AAA TGC AGG T
	Reverse	CAG GGC CAT GCG GCT TCT
Control site	Forward	AAA AAC GGG GTC AGA AGT CAG GAA
	Reverse	AGG TAA AGA TTG GGG AAG CAG CAA

Supplementary Table S9. RNA-sequencing quality assessment.

Sample	RNA integrity number	Raw reads	Clean reads	Uniquely mapped reads (%)	Error rate (%)	GC Content (%)
Vector	8.6	32577966	31766547	91.86	0.03	50.40
TCF7L1	9.4	22844683	22344422	92.51	0.03	50.27
TCF7L1^{MUT}	9.4	26703646	26033154	92.38	0.03	49.75

Supplementary Table S10. ChIP-sequencing quality assessment

Summary of raw data quality control

Sample	Raw reads	Low quality reads	Degenerative reads	Empty reads	Too short reads	Clean reads	Clean rate (%)
TCF7L1	19620917	445	0	0	3	19620469	100.00
TCF7L1^{MUT}	20164649	518	0	0	4	20164127	100.00

Note: Low quality reads are reads with mean quality lower than 20 before trimming. Degenerative reads are reads with at least 15% "N" before trimming. Empty reads are reads with all bases from adapter. Too short reads are reads shorter than 18 nt that are discarded after trimming. Clean reads are reads kept after trimming. Clean rate is the ratio of clean reads to raw reads.

Summary of mapping

Sample	Reads	Clean reads	Mapped (relative to clean reads)
TCF7L1	Pair	19620469	18970792(96.69%)
TCF7L1	Read1	19620469	19606934(99.93%)
TCF7L1	Read2	19620469	19606641(99.93%)
TCF7L1^{MUT}	Pair	20164127	19684614(97.62%)
TCF7L1^{MUT}	Read1	20164127	20150536(99.93%)
TCF7L1^{MUT}	Read2	20164127	20150451(99.93%)

Summary of fragment size

Sample	Fragment Size ± Standard Deviation
TCF7L1	310 bp ± 100 bp
TCF7L1^{MUT}	299 bp ± 92 bp

Summary of strand cross correlation

Sample	Normalized strand coefficient (NSC)	Relative strand correlation (RSC)
TCF7L1	1.0913	2.2955
TCF7L1^{MUT}	1.079	2.0761

Summary of peak calling

Sample	Count of narrow peaks	Fraction of reads in peaks (FRiP)	Count of summits
TCF7L1	3663	1.0447%	3762
TCF7L1^{MUT}	204	0.2859%	224
